# Complete mitochondrial genome and phylogenetic analysis of *Ixodes acutitarsus* (Acari: Ixodidae)

**DOI:** 10.1080/23802359.2022.2089066

**Published:** 2022-06-22

**Authors:** Xinyan Lu, Dandan Jiang, Chunhong Du, Caihong Rao, Jianqiang Yin, Yihao Fang, Xing Yang

**Affiliations:** a Integrated Laboratory of Pathogenic Biology, College of Preclinical Medicine, Dali University, Dali, P. R. China; b School of Public Health, Dali University, Dali, P. R. China; c Endemic Diseases Control and Prevention, Yunnan Institute of Endemic Diseases Control and Prevention, Dali, China; d HighliGongshan National Nature Reserve Fugong Management and Protection Branch, Nujiang Lisu Autonomous Prefecture, Yunnan, China; e Institute of Eastern-Himalaya Biodiversity Research, Dali University, Dali, Yunnan, China

**Keywords:** *Ixodes acutitarsus*, complete mitogenome, phylogeny

## Abstract

*Ixodes acutitarsus* is regarded as the largest Ixodes tick around the world. *I. acutitarsus* is capable to transmit a wide range of animal and human pathogens. This research pioneered sequencing of the complete mitochondrial genome of *I. acutitarsus*. With a length of 14,475 bp, the complete mitochondrial genome encodes 13 protein-coding genes (PCGs), two ribosomal RNA genes (rRNAs), 22 transfer RNA genes (tRNAs), and one replication-initiating region. The phylogenetic relationship was established using the Maximum-likelihood method to indicate that *I. acutitarsus* and the others of the genus *Ixodes* fit into the same branch, which confirms the inclusion of *I. acutitarsus* in the genus *Ixodes.* The complete mitogenome of sequenced *I. acutitarsus* provides molecular evidence for the taxonomic status and phylogenetic position of several *Ixodes* species.

The obligate hematophagous arthropod *Ixodes acutitarsus* (Karsch, 1880) (Acari: Ixodidae) is widely distributed in many Asian countries and regions, including Southwest China, Nepal, Taiwan, Burma, Japan, and India (Daniel 1979). As the principal transmission vector, *I. acutitarsus* acts as the reservoir of various tick-transmitted pathogens that include a virus, bacteria, and protozoa (Chao and Shih 2012). However, there remains a considerable gap in molecular epidemiology and genetics owing to the lack of appropriate genetic markers for these ticks (Shao et al. 2005). Up to now, there is only a single ‘unverified’ (and lacking accurate genetic annotation information) mitochondrial sequence of *I. acutitarsus,* which is a linear sequence with a size of 14,484 bp.

Herein, the first complete mitochondrial genome of *I. acutitarsus* was sequenced, annotated, and verified. Adult ticks (three male and three females) were collected in February 2021 from High Li Gong Shan, Fugong county-level town, Nujiang Lisu Autonomous Prefecture, northwest Yunnan Province, China (26°34′N, 98°48′E). Species identification was performed by Professor Chunhong Du according to the exact morphological characteristics (Lu et al. 2021). One male and one female were treated as voucher specimens and the remaining ones were used for DNA extraction. Then, the collected specimens were stored in the Parasitological Museum, Dali University (Yunnan, China) under the collection number: DLUP2102_11-12 (Url: http://www.dali.edu.cn/jcyxy/xkpt/jcyxsyjxzx/6431.htm, Contact person: Xing Yang, yang08220013@163.com) (Lu et al. 2021). The circle genomics DNA was isolated using the standard CTAB technique and then preserved in 75% ethanol at −20 °C. The complete mitochondrial genome was sequenced on the Illumina NovaSeq platform (Shanghai Personal Biotechnology Co, Ltd, Shanghai, China), assembled using A5-miseq software (Coil et al. 2015), and annotated using the MITOS web server (http://mitos.bioinf.uni-leipzig.de/) (Bernt et al. 2013).

The annotated complete mitochondrial genome of *I. acutitarsus* is 14,475 bp in size (GenBank accession number: OL800704) with 37 genes, including 13 PCGs, 2 rRNAs, 22 tRNAs, and 1 replication-initiating region. The total base content of the *I. acutitarsus* mitochondrial genome is 40.01% T, 13.99% C, 38.31% A, and 7.7% G. The size of *I. acutitarsus* small subunit rRNA and large subunit rRNA was 723 bp and 1,245 bp, respectively. Four of these PCGs (*NAD1, NAD5, NAD4L,* and *NAD4*) were encoded by the light strand (L-strand), while all of the remaining PCGs (*COX1, NAD2, COX2, ATP8, COX3, ATP6, NAD3, CYTB, NAD6*) were encoded by the heavy strand (H-strand). The length of the 22 tRNAs varies from 56 bp (tRNA-Ser) to 68 bp (tRNA-Gln), with fourteen tRNA genes encoded on the H-strand (Lowe and Chan 2016). Among thirteen PCGs, *NAD4L, COX3, ATP6, COX2, NAD1, NAD4, CYTB,* and *NAD3* start with ATG, *NAD2, NAD6, ATP8* start with ATA, and the remaining two PCGs use ATT as the start codon. In addition, most genes use TAA as the stop codon, while *COX3, COX2, CYTB, NAD1,* and *NAD5* use the incomplete termination codon T, *NAD4L* terminate with TAG.

An alignment of published complete mitogenomes of Ixodidae species was constructed with the 13 PCGs together with Limulus polyphemus (NC_003057) as an outgroup. A phylogenetic tree was then obtained applying a Maximum-likelihood analysis implemented in MEGA7.0 software (Figure 1) (Kumar et al. 2016).

**Figure 1.**
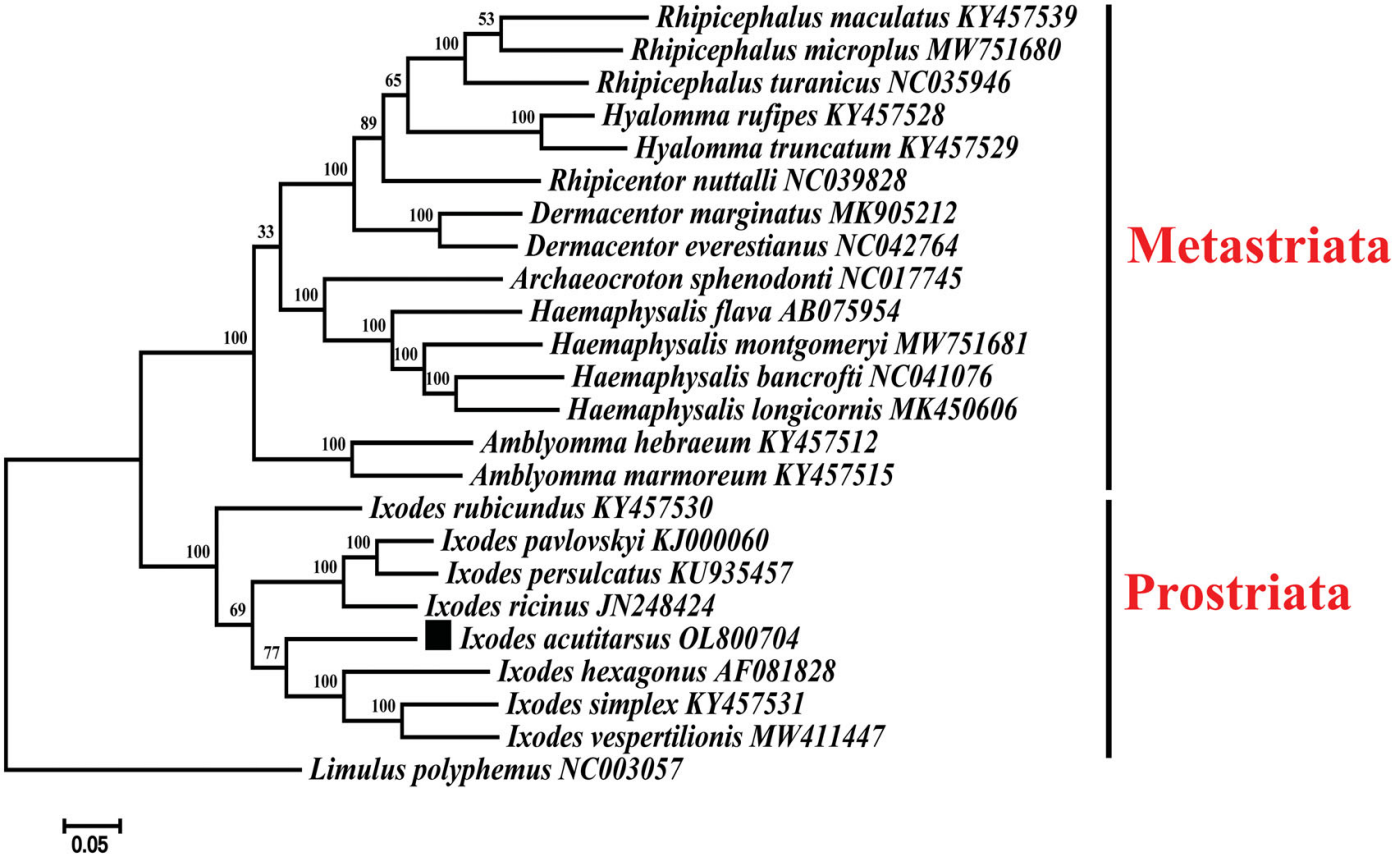
Maximum-likelihood (ML) phylogeny of 23 species of the family Ixodidae based on the 13 concatenated nucleotide sequences of protein-coding genes (PCGs), utilizing GTR + G + I model and after 1,000 bootstrap replications. The black square sign represents the species in this study. Bootstrap support values are shown above the nodes.

The phylogenetic tree show two phylogroups: Metastriata and Prostriata (Cummings et al. 1995). The first branch includes species of seven genera, namely, *Rhipicephalus, Hyalomma, Rhipicentor, Dermacentor, Amblyomma, Archaeocroton, Haemaphysalis*. The second branch includes only the species of the genus *Ixodes*. *Ixodes acutitarsus* clusters within the *Ixodes* clade. Besides, the species showed a closer relationship with *I. hexagonus,* followed by *I. simplex*, *I. vespertilionis*, *I. ricinus*, *I. persulcatus*, and *I. pavlovskyi*. The sequences have only 61.01% similarity to unconfirmed sequences, but more than 90% similarity to confirmed partial sequences. The sequences as measured in this study were found to be identical to some of the published *I. acutitarsus* genes in NCBI, despite a low-level resemblance to the unconfirmed complete sequence data. In conclusion, the new complete mitochondrial genome of *I. acutitarsus* is an important resource to enhance future phylogenetic studies within *Ixodidae*. (Tao et al. 2014).

## Data Availability

The data that support the findings of this study are openly available in the National Center for Biotechnology Information (NCBI) at https://www.ncbi.nlm.nih.gov. The accession number of the complete mitochondrial genome is OL800704. The associated BioProject, SRA, and Bio-Sample numbers are PRJNA807826, SRR18056297 and SAMN26001839, respectively.
